# Urological Injuries Following Gynecologic and Obstetric Surgery: Incidence, Diagnosis, and Outcomes from a 10-Year Retrospective Cohort

**DOI:** 10.3390/healthcare14030327

**Published:** 2026-01-28

**Authors:** Eser Ördek, Ahmet Beyazıt, Sadık Görür, Kenan Dolapçıoğlu, Fatih Gökalp, Nezih Tamkaç

**Affiliations:** 1 Department of Urology, Faculty of Medicine, Hatay Mustafa Kemal University, 31060 Hatay, Türkiye; 2 Department of Obstetrics and Gynecology, Faculty of Medicine, Hatay Mustafa Kemal University, 31060 Hatay, Türkiye

**Keywords:** iatrogenic urinary tract injuries, gynecological and obstetric surgery, early diagnosis and surgical management

## Abstract

Background/Objectives: Gynecological and obstetric surgeries carry a risk of iatrogenic urinary tract injuries; however, comparative data on injury patterns, diagnostic timing, and management across different surgical indications remain limited. This study aimed to evaluate the incidence, characteristics, diagnostic timing, and outcomes of urological injuries following gynecologic and obstetric surgeries in a high-volume tertiary referral center over a 10-year period. Methods: This retrospective single-center cohort study included adult female patients who sustained intraoperative or postoperative urological injuries during gynecologic or obstetric procedures between January 2014 and December 2024. Urological injury was defined as bladder, ureteral, or genitourinary fistula injury requiring urological intervention. Patients with prophylactic or temporary ureteral stenting were excluded. Cases were classified into malignant gynecologic, obstetric, and benign gynecologic surgery groups. Injury type, timing of diagnosis, management strategies, and clinical outcomes were analyzed. Results: Among 16,100 procedures, 223 urological injuries were identified (incidence: 1.3%). Bladder injuries were the most common (62.3%) and were predominantly associated with obstetric procedures, whereas ureteral injuries (28.7%) occurred more frequently during malignant gynecologic surgeries (*p* < 0.05). Intraoperative recognition rates varied significantly by injury type, being highest for bladder injuries (98.6%) and lowest for fistulas (5.0%) (*p* < 0.001). Conclusions: Surgical indication significantly influences the pattern and timing of urological injuries. Bladder injuries are usually detected intraoperatively, whereas ureteral and fistulous injuries are more often diagnosed postoperatively, particularly in malignant and complex procedures, highlighting the need for targeted intraoperative vigilance and multidisciplinary management in high-risk cases.

## 1. Introduction

The shared embryological origin and anatomical proximity of the urogenital system contribute to the relatively high incidence of iatrogenic urinary tract injuries during obstetric and gynecological surgeries [1]. Despite advances in surgical techniques, such injuries remain a clinically relevant problem. Owing to the complex pelvic anatomy, approximately 75% of iatrogenic urinary tract injuries occur during gynecological procedures, with reported incidence rates of 0.5–1% in pelvic and abdominal surgeries, most commonly involving the bladder [2,3].

Urinary tract injuries may manifest as bladder perforation, ureteral ligation or avulsion, and genitourinary fistula formation. These complications are influenced by multiple factors, including surgical technique, prior operations with adhesions, anatomical distortion caused by malignancy or deep infiltrative pathology, and emergency surgical conditions [4]. While early recognition and timely intervention reduce morbidity and help preserve quality of life, delayed diagnosis may result in severe complications such as ureterovaginal or vesicovaginal fistulas, leading to prolonged morbidity [5].

Although several studies have examined urinary tract injuries in specific surgical settings, comparative data on injury patterns, diagnostic timing, and management outcomes across malignant, obstetric, and benign gynecological surgeries within the same clinical environment remain limited. Therefore, this study provides a 10-year, single-center evaluation of urogenital injuries following obstetric and gynecological surgeries, focusing on differences according to surgical indication.

## 2. Methods

### 2.1. Participants

Between January 2014 and December 2024, a total of 16,100 gynecologic and obstetric surgical procedures were performed at our institution. Among these procedures, 223 female patients who developed urogenital injuries and were treated at the Department of Urology were retrospectively identified and included in the study. The injuries were identified from a wide range of procedures, including cesarean sections, benign hysterectomies, and malignant gynecologic surgeries. The risk of urinary tract injury differed according to the surgical type, being most frequently associated with cesarean section and hysterectomy procedures. Eligible participants were women aged 18 years or older who underwent surgery for either benign or malignant indications and subsequently experienced intraoperative urinary tract injury. Patients who underwent prophylactic or temporary ureteral stent placement were excluded from the analysis. Patient-related risk factors such as previous pelvic surgery, radiotherapy, pelvic inflammatory disease, or deep endometriosis were not analyzed separately and were not used as exclusion criteria. All included cases represent patients directly treated and followed at our institution; injuries diagnosed and managed at outside hospitals were not included in this study.

### 2.2. Study Design

Patients were divided into three groups based on surgical indications: The first group included malignant gynecologic surgeries, the second comprised obstetric procedures such as cesarean sections, and the third consisted of benign gynecologic surgeries, including myomectomies and hysterectomies. Group classification based on surgical indication was predefined to reflect clinically distinct operative settings with different levels of surgical complexity and risk of urological injury. The groups were evaluated with respect to the occurrence of urologic complications and the treatment modalities applied. In addition, cases were categorized as bladder/ureteral injuries or genitourinary fistulas according to the type of injury, and subgroup analyses were performed regarding time of diagnosis, therapeutic approach, and clinical outcomes. Clinical data—including patient age, surgical indication and procedure type, injury site and characteristics, duration of hospitalization, catheterization time, and timing of diagnosis and treatment—were retrieved from medical records. In this retrospective descriptive cohort study, the primary outcomes were type of urological injury and timing of diagnosis. Secondary outcomes included management strategies and clinical course, while patient characteristics and surgical details were analyzed as descriptive variables. Urological complications were defined as genitourinary tract injuries detected intraoperatively or requiring postoperative intervention. Diagnostic evaluation consisted of physical examination, urogynecological assessment, and imaging studies. Complications and management strategies were analyzed in line with current literature. The study was approved by the Ethics Committee of Hatay Mustafa Kemal University (authorization number: 02/10/2024/03) and conducted in accordance with the principles of the Declaration of Helsinki.

### 2.3. Statistical Analysis

Statistical analyses were performed using SPSS version 22. Continuous variables were summarized using mean ± standard deviation or median (minimum–maximum), and categorical variables as frequencies and percentages. Group comparisons were conducted using the Mann–Whitney U test or Kruskal–Wallis test for continuous variables and the chi-square or Fisher’s exact test for categorical variables, as appropriate. All intergroup comparisons were exploratory and descriptive in nature, aimed at identifying potential patterns rather than testing predefined hypotheses; therefore, no adjustment for multiple comparisons was applied. A *p*-value < 0.05 was considered statistically significant.

## 3. Results

**Figure 1:**
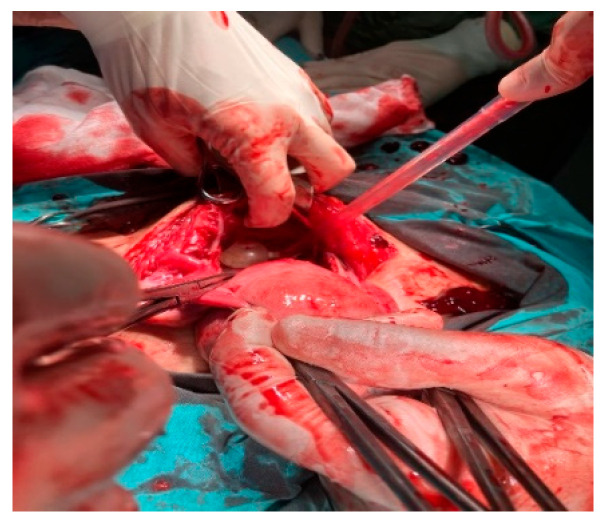
Intraoperative image showing bladder perforation and its open surgical repair.

The study included 223 women with a mean age of 48 years (range, 22–93 years). The most common procedures were total abdominal hysterectomy (35.4%) and cesarean section (26.5%), followed by total laparoscopic hysterectomy and cesarean section with peripartum hysterectomy. Overall, bladder injuries were the most frequent type (62.3%), followed by ureteral injuries (28.7%) and genitourinary fistulas (9.0%). Most urological injuries (81.6%) were diagnosed intraoperatively, predominantly through direct visual inspection (Table 1, Figure 1).

Among patients with fistulas, 55.0% had undergone benign hysterectomy, whereas among those with ureteral injury, 50.0% had undergone malignant hysterectomy (*p* < 0.001). Across all groups, complication rates were higher following open surgery (*p* = 0.026) (Table 2).

**Figure 2:**
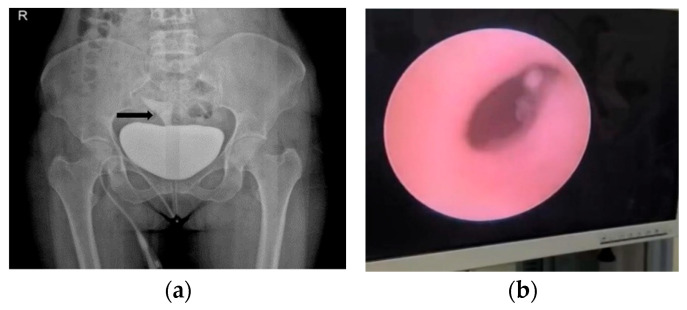
Representative images of urological injuries following gynecologic surgery. (**a**), vesicouterine fistula. (**b**), ureteral avulsion (endoscopic image).

Bladder injuries were managed with surgical repair using a double-layer suture technique in all cases. Ureteral injuries were predominantly treated with ureteroneocystostomy, while percutaneous nephrostomy was applied when clinically indicated. Among genitourinary fistulas, vesicovaginal fistula repair was the most frequently performed procedure. Intraoperative detection rates differed markedly according to injury type, being highest for bladder injuries and lowest for fistulas (Figure 2a,b).

The duration of Foley catheterization differed between injury groups, with the longest mean duration observed in the fistula group (22.3 days) and the shortest in the ureteral injury group (10.1 days). Similarly, the time to surgical repair varied among injury types, being shortest for bladder injuries and longest for fistulas (Table 2).

The mean retention period of double-J stents was longer in Group 1 (47.8 days) compared to Groups 2 (42.1 days) and 3 (41.2 days). Foley catheter indwelling time was longest in Group 2 (17.3 days). Mortality was observed exclusively in Group 1 (Table 3).

Postoperative symptoms showed significant intergroup variation (*p* < 0.001). The highest rate of asymptomatic patients was in Group 2, while Group 1 demonstrated higher frequencies of flank pain, dysuria, sepsis (27.0%), and fever (39.7%) (Table 3).

## 4. Discussion

Urinary tract injuries occurring during gynecological and obstetric surgeries represent a common urological complication of pelvic surgery, and delayed diagnosis or treatment may increase morbidity and impair quality of life [6]. Reported incidence rates range from 0.3% to 1.5%, with bladder injuries being more frequent than ureteral injuries [3]. Despite advances in surgical techniques, these injuries remain clinically relevant. In the present study, the observed incidence of 1.3% lies toward the upper range of values reported in the literature and may be related to procedural characteristics and intraoperative conditions rather than a single causative factor [7].

In line with previous reports, bladder injuries constituted the majority of cases, followed by ureteral injuries and genitourinary fistulas, reflecting the anatomical vulnerability of the bladder and distal ureter during pelvic surgery. Similar distributions have been reported in the literature, with bladder injury being the most common complication, whereas ureteral injuries, although less frequent, are often associated with greater morbidity [8]. Genitourinary fistulas generally represent a delayed manifestation of injuries that are missed intraoperatively or diagnosed postoperatively [9].

Early recognition of iatrogenic urinary tract injuries is crucial for reducing morbidity. In the present study, most lesions were identified intraoperatively, highlighting the importance of surgical awareness and anatomical knowledge; however, a substantial proportion of injuries were diagnosed postoperatively, suggesting that ureteral injuries and fistulas are more likely to be overlooked during surgery. Similar observations have been reported in the literature, where only a minority of ureteral injuries are recognized intraoperatively, with many becoming clinically evident within 48–72 h due to nonspecific symptoms [10].

The timing of diagnosis appears to be closely related to the type of injury. Bladder injuries are generally recognized during surgery, whereas ureteral injuries and fistulas are more frequently diagnosed postoperatively, likely reflecting the subtle nature of these lesions and delayed clinical presentation. Fistulas are often detected at a later stage, presenting with symptoms such as pelvic pain, urinary incontinence, or cyclic hematuria [11]. In high-risk procedures, intraoperative cystoscopy and visualization of ureteral urine jets may assist in earlier detection and improved outcomes. These practices are recommended by the AUA, and increased familiarity with cystoscopy among gynecologic surgeons may facilitate timely recognition and management of urinary tract injuries [12,13].

The type of urinary tract injury varies according to surgical indication and operative approach. In this series, fistula formation was more frequently observed after hysterectomy performed for benign pathology, which may be related to increased dissection difficulty in the presence of chronic inflammation and adhesions [14]. In malignant cases, the higher rate of ureteral injuries likely reflects the need for more extensive dissections and possible parametrial involvement. Higher complication rates observed in open procedures further suggest that both surgical indication and approach influence the risk of urological injury, underscoring the importance of individualized surgical planning [15]. However, it should be noted that the choice of surgical approach in this cohort was primarily determined by underlying pathology and urgency rather than random allocation.

In the present study, a substantial proportion of bladder injuries exceeded 2 cm in size, a threshold recognized as a clinically relevant marker for predicting technical difficulty and postoperative complications [16]. Larger bladder defects are particularly relevant in procedures requiring extensive bladder dissection, such as abdominal hysterectomy, where surgical expertise and anatomical knowledge are essential. Such defects may increase the complexity of repair, prolong catheterization, and elevate the risk of fistula formation [17]. In our cohort, all bladder injuries were repaired using a double-layer suture technique in accordance with established recommendations, while larger defects may require more advanced reconstructive strategies to optimize outcomes.

The localization and type of ureteral injuries indicate that certain segments are particularly vulnerable during pelvic surgery. In this study, most ureteral injuries involved the distal ureter, likely reflecting its susceptibility during uterine artery ligation or pelvic wall dissection [10]. Thermal injury constituted a notable proportion of cases, emphasizing the need for cautious use of energy devices, especially in laparoscopic procedures, as such injuries may remain unrecognized intraoperatively and later present as strictures or fistulas [18]. In selected high-risk cases, intraoperative cystoscopy or assessment of ureteral urine jets may facilitate earlier detection.

Ureteral injuries were most commonly managed with ureteroneocystostomy and double-J stent placement, while percutaneous nephrostomy was reserved for selected patients, particularly those with delayed diagnosis or increased risk of infection. In such cases, PCN plays an important role in preserving renal function and ensuring adequate urinary drainage. Similar benefits of PCN in postoperatively diagnosed ureteral injuries have been reported in the literature [19]. In selected cases of ureteral injury, ureteral catheter or stent placement under direct laparotomic or laparoscopic guidance has also been described as a rapid, safe, and cost-effective alternative that avoids radiation exposure. This approach may facilitate intraoperative management, particularly in centers with appropriate surgical expertise, and has been increasingly adopted in recent clinical practice [20].

In the fistula group, vesicovaginal fistula repair was the predominant surgical approach, consistent with previous reports [21]. Less frequent fistulas included vesicouterine and vesicorectal types, which are typically associated with obstetric complications or extensive pelvic surgery and often present with delayed or complex clinical manifestations [22]. The success of fistula repair depends on several local and patient-related factors; therefore, optimal timing of intervention and individualized management within a multidisciplinary framework remain essential.

The rate and duration of double-J stent placement in ureteral injuries reflect both lesion severity and therapeutic strategy. In the present study, stents were most commonly placed unilaterally, with a mean indwelling time that exceeded the recommended 4–6 weeks, potentially influenced by factors such as patient compliance and institutional workload [23]. Prolonged stent duration and higher stent utilization were more frequently observed in patients undergoing malignant surgery, suggesting a greater complexity of periureteral involvement in this group.

These observations may be related to the extensive dissections required in advanced malignancies, distortion of pelvic anatomy, and increased perioperative risk. Consistent with previous reports, malignant pelvic surgeries have been associated with higher rates of ureteral injury and morbidity compared with benign or obstetric procedures, particularly in the presence of parametrial extension [19]. Postoperative inflammation, delayed tissue healing, and adjuvant treatments may further contribute to prolonged stent requirements in malignant cases. Accordingly, careful preoperative risk assessment, intraoperative urological collaboration, and individualized postoperative follow-up remain important for optimizing outcomes.

Patients with bladder and ureteral injuries demonstrated different Foley catheterization durations compared with those in the fistula group, with the longest duration observed among patients with fistulas. Prolonged catheterization in fistula cases is generally required to ensure adequate bladder rest and optimal tissue healing. For vesicovaginal and vesicouterine fistulas, continuous bladder drainage for 14–21 days has been recommended to improve surgical outcomes [24]. In contrast, catheterization duration following isolated bladder injury may be shorter, although reported practices vary, and some studies suggest that drainage periods as short as 7 days may be sufficient in selected cases [25]. These observations indicate that catheterization duration should be individualized according to injury type and complexity. It should also be noted that catheter and stent management in the present series largely reflects institutional practice and may therefore differ from approaches used at other centers.

Postoperative clinical symptoms varied according to surgical etiology. Patients undergoing obstetric procedures were more frequently asymptomatic, whereas symptomatic presentations were more common following malignant surgery. In particular, flank pain and dysuria were observed more often in the malignant surgery group, which may be related to the invasive nature of oncologic procedures and the extent of periureteral dissection [26]. In addition, postoperative fever and sepsis were more prevalent among patients undergoing malignant surgery, possibly reflecting longer operative times, greater surgical trauma, and immunosuppression in advanced disease. These findings underscore the need for closer postoperative surveillance in this patient population. Similar trends have been reported in the literature, with higher rates of infectious complications following pelvic surgery for malignancy compared with benign or obstetric indications [27]. The present findings are consistent with this evidence and further support the increased postoperative risk associated with malignant gynecologic surgery.

In complex gynecological malignancy surgeries, factors such as fibrosis, prior radiotherapy, and distorted pelvic anatomy substantially increase the risk of ureteral injury. For this reason, the use of prophylactic double-J stents has been proposed in selected high-risk cases. However, the literature remains inconclusive, as some studies report improved intraoperative recognition, whereas others suggest that surgical experience and meticulous dissection are more decisive than routine stenting [28,29]. Accordingly, prophylactic stenting is not routinely recommended and should be reserved for carefully selected patients. In the present study, no patients underwent preoperative stenting, underscoring the need for prospective studies to better define its role. In addition, preoperative urological consultation was infrequently performed, despite evidence that multidisciplinary collaboration may reduce complications through earlier recognition and optimized management [30]. These findings support a selective approach to prophylactic stenting and highlight the importance of multidisciplinary strategies in high-risk malignancy surgeries.

### Limitations

This study is limited by its retrospective, single-center design, which may introduce information bias and restrict generalizability. The high surgical volume of our tertiary referral center and the lack of routine preoperative urological consultation may have contributed to delayed recognition in a minority of cases. In addition, patient- and disease-related factors such as prior radiotherapy, pelvic fibrosis, deep infiltrating endometriosis, or complex inflammatory conditions—known to increase the risk of urological injury—were not analyzed separately and may have influenced injury occurrence. These factors warrant further evaluation in future prospective studies. Despite these limitations, this 10-year analysis provides valuable real-world data on the management and outcomes of urological injuries following gynecological and obstetric surgeries.

## 5. Conclusions

Urological injuries following gynecological and obstetric surgeries demonstrate distinct patterns depending on surgical indication and injury type. In this cohort, bladder injuries were predominantly recognized intraoperatively, whereas ureteral injuries and fistulas were more frequently diagnosed postoperatively, particularly in malignant and complex procedures. These findings highlight that timing of diagnosis and injury characteristics differ substantially between malignant, obstetric, and benign surgeries, underscoring the need for heightened intraoperative awareness in high-risk settings. Overall, this 10-year single-center experience provides clinically relevant insights into injury patterns and diagnostic timing, and suggests that coordinated perioperative management may help reduce morbidity in selected high-risk cases.

## Figures and Tables

**Table 1:** General characteristics of patients.

Variables	*n* = 223
Age (years), Mean (Min–Max)	48 (22–93)
Vaginal delivery, Mean (Min–Max)	3.5 (1–13)
Cesarean Section, Mean (Min–Max)	2.6 (1–6)
TAH, *n* (%)	79 (35.4)
TLH, *n* (%)	34 (15.2)
C/S, *n* (%)	59 (26.5)
C/S peripartum hysterectomy, *n* (%)	42 (18.8)
C/S uterine rupture repair, *n* (%)	5 (2.2)
Pop surgery, *n* (%)	4 (1.8)
Bladder injury, *n* (%)	139 (62.3)
Ureteral injury, *n* (%)	64 (28.7)
VUF, VVF, VRF, *n* (%)	20 (9.0)
Bladder Injury Size, *n* (%)	
≤2 cm	52 (37.4)
>2 cm	87 (62.6)
Ureteral Injury Location, *n* (%)	
Distal	48 (75.0)
Middle	16 (25.0)
Ureteral Injury Type, *n* (%)	
Thermal	16 (25.0)
Complete	16 (25.0)
Partial	32 (50.0)
Recognition Time, *n* (%)	
Intraoperative	182 (81.6)
Postoperative	41 (18.4)
Postoperative Repair Time (days), Mean (Min–Max)	68.4 (5–180)
Diagnostic Techniques, *n* (%)	
Direct visual	182 (81.6)
Histogram	1 (0.4)
CT urogram	20 (9.0)
Cystogram + CT urogram	20 (9.0)
Exitus, *n* (%)	3 (1.3)

Pop surgery: pelvic organ prolapse surgery, CT urogram: computer tomography urogram.

**Table 2:** Comparison of bladder, ureter, and fistula injuries.

Variables	Bladder Injury (*n* = 139)	Ureter Injury (*n* = 64)	Fistula Damage (*n* = 20)	*p* Value
	*n* (%)	*n* (%)	*n* (%)	
Hysterectomy (Etiological)				
Benign	27 (19.4)	16 (25.0)	11 (55.0)	* 0.002
Malignant	27 (19.4)	32 (50.0)	4 (20.0)	* <0.001
C/S peripartum hysterectomy	30 (21.6)	9 (14.1)	3 (15.0)	* 0.400
Hysterectomy (Surgical Technique)				
Laparoscopic surgery	17 (12.2)	16 (25.0)	1 (5.0)	* 0.026
Open surgery	122 (87.8)	48 (75.0)	19 (95.0)	
C/S	50 (36.0)	7 (10.9)	2 (10.0)	* <0.001
C/S uterine rupture repair	5 (3.6)	0 (0.0)	0 (0.0)	* 0.454
Pop surgery	4 (2.9)	0	0	* 0.528
Bladder Injury Management				
Bladder repair (double-layer)	139 (100.0)	0	0 (0.0)	-
Ureteral Injury Management				
UNC (right/left)	0 (0.0)	48 (75.0)	0 (0.0)	-
Bilateral UNC	0	14 (21.9)	0	-
Ureteroureterostomy	0 (0.0)	2 (3.1)	0	-
Fistula Management				
VUF repair	0 (0.0)	0	1 (5.0)	-
VVF repair	0	0	18 (90.0)	-
VRF repair	0	0 (0.0)	1 (5.0)	-
Recognition Time				
Intraoperative	137 (98.6)	44 (68.8)	1 (5.0)	* <0.001
Postoperative	2 (1.4)	20 (31.3)	19 (95.0)	
PCN				
Present	0 (0.0)	17 (26.6)	0 (0.0)	-
Absent	0	47 (73.4)	0 (0.0)	-
DJ				
Bilateral	0 (0.0)	14 (21.9)	0 (0.0)	* <0.001
Unilateral	0	49 (76.6)	0 (0.0)	
None	139 (100.0)	1 (1.6)	20 (100.0)	
Postoperative Repair Time (days), Mean (Min-Max)	-	*n* = 20 54.2 (10–120)	*n* = 20 90 (60–180)	*** 0.001
Length of Foley Catheterization (days), Mean (Min-Max)	17.4 (1–30)	10.1 (1–30)	22.3 (14–45)	** <0.001 ^a,b,c^
DJ Stent Retention Period (days), Mean (Min-Max)	-	44.7 (30–90)	-	-

* Pearson Chi-Square Test or Fisher’s Exact Test, ** Kruskal–Wallis H Test, *** Mann–Whitney-U Test. ^a^: Significant difference between bladder and ureter injury, ^b^: Significant difference between bladder and fistula injury, ^c^: Significant difference between ureter and fistula injury.

**Table 3:** Intergroup comparisons.

Variables	Group 1 Malignant (*n* = 63)	Group 2 Obstetric (*n* = 106)	Group 3 Benign (*n* = 54)	*p* Value
Postoperative Symptoms, *n* (%)				
None	35 (55.6)	96 (90.6)	35 (64.8)	* <0.001
Dysuria	5 (7.9)	2 (1.9)	1 (1.9)	
Side pain	19 (30.2)	4	5 (9.3)	
Hematuria	0 (0.0)	0 (0.0)	2 (3.7)	
Sepsis, *n* (%)	17 (27.0)	2 (1.9)	3 (5.6)	* <0.001
Fever, *n* (%)	25 (39.7)	10 (9.4)	6 (11.1)	* <0.001
Postoperative bilateral DJ, *n* (%)	11 (17.5)	2 (1.9)	2 (3.7)	* <0.001
Postoperative unilateral DJ, *n* (%)	22 (34.9)	14 (13.2)	13 (24.1)	* 0.004
Postoperative PCN, *n* (%)				
Present	9 (28.1)	3 (18.8)	5 (31.3)	* 0.811
Absent	23 (71.9)	13 (81.3)	11 (68.8)	
DJ stent retention period (days), Mean (Min–Max)	47.8 (30–90)	42.1 (30–90)	41.2 (30–60)	** 0.039
Length of Foley catheterization (days), Mean (Min–Max)	13.9 (5–28)	17.3 (1–30)	14.7 (1–45)	** <0.001 ^a,b^
Hospital stay (days), Mean (Min–Max)	9.5 (1–35)	7.1 (2–35)	6.1 (1–60)	** <0.001 ^a,b,c^
Exitus, *n* (%)	3 (4.8)	0 (0.0)	0 (0.0)	* 0.035

^a^: Significant difference between Group 1 and Group 2, ^b^: Significant difference between Group 2 and Group 3, ^c^: Significant difference between Group 1 and Group 3, * Pearson Chi-Square Test or Fisher’s Exact Test, ** Kruskal-Wallis H Test.

## Data Availability

The data presented in this study are available on request from the corresponding author due to privacy restrictions.

## Abbreviations

IBI	iatrogenic bladder injury
IUI	iatrogenic urinary injuries
TAH	total abdominal hysteroctomy
TLH	total laparoscopic hysteroctomy
C/S	caesarean section
POP	pelvic organ prolapse
VUF	vesicouterine fistula
VVF	vesicovaginal fistula
VRF	vesicorectal fistula
UNC	ureteroneocystostomy
PCN	percutaneous nephrostomy
DJ	double j
AUA	American Urological Association
